# P-2181. Cardiac MRI Identifies Heart Disease Risk in Individuals with Hepatitis C Regardless of Myocardial Damage Markers or Fibrosis Stage

**DOI:** 10.1093/ofid/ofae631.2335

**Published:** 2025-01-29

**Authors:** Aaron D’Amore, Arlene Sirajuddin, Nivya George, Ahmed M Gharib, Poonam Mathur

**Affiliations:** Harvard - Mass General Brigham, Cambridge, Massachusetts; National Institutes of Health, Bethesda, Maryland; St. Jude Children’s Research Hospital, Memphis, Tennessee; National Institutes of Health, Bethesda, Maryland; University of Maryland, Baltimore, Maryland

## Abstract

**Background:**

Infection with hepatitis C virus (HCV) increases the risk of extrahepatic manifestations, including cardiovascular disease (CVD). Cardiac magnetic resonance (CMR) remains the gold standard test for non-invasive structural and functional assessment of the heart and can be used to detect CVD. We sought to determine the CVD risk in patients with HCV prior to therapy using CMR.

**Table 1.**
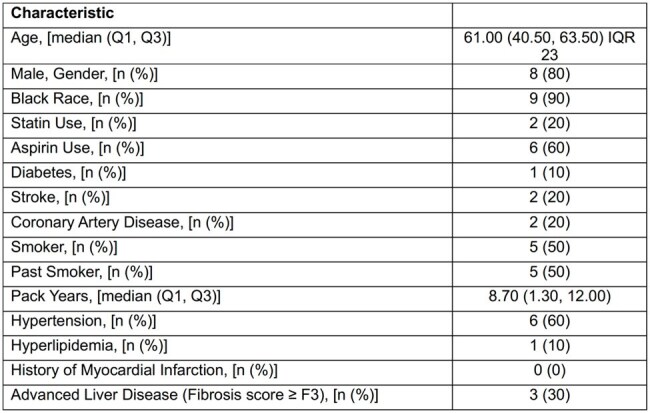
Baseline Demographic Features of HCV CMR Cohort

**Methods:**

Individuals with chronic HCV infection were prospectively enrolled in the CHROME study (NCT 03823911). A subset of 10 HCV-infected individuals were enrolled in the MRI sub-study from March to September 2019. HCV antibody and RNA, liver fibrosis score, and markers of inflammation and myocardial damage were obtained.

CMR was performed prior to the initiation of DAA treatment. ECV fraction was calculated using the equation: ECV = 100% x (1-hematocrit) x [(1/T1myocardium post) – (1/T1myocardium pre)]/[(1/T1blood post) – (1/T1 blood pre)]. An ECV fraction > 30% was considered abnormally increased. 10 age-matched, HCV-negative volunteers were included in the analysis so that T-test was used to compare ECV fraction in both groups.

**Figure 1.**
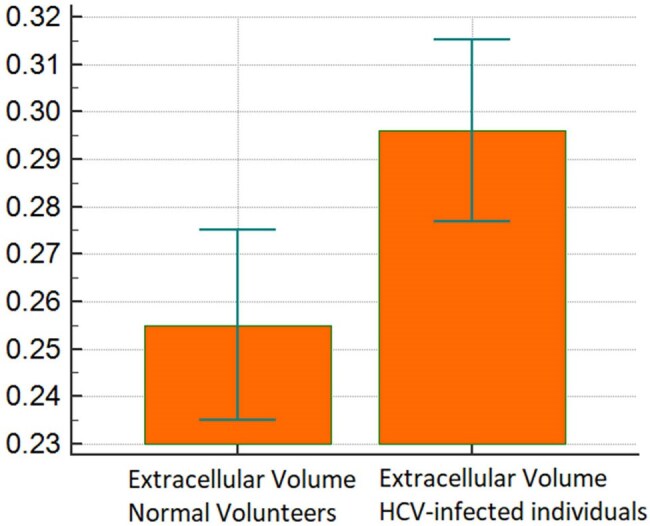
Extracellular Volume (ECV) of HCV-infected individuals and Healthy Volunteers. ECV fraction in the HCV-infected individuals was significantly increased (0.30 ±0.03 vs. 0.26±0.03, p=0.0036).

**Results:**

Demographics are shown in Table 1. CMR showed that 8 of 10 HCV-infected individuals had an abnormal ECV fraction ( >30%)^7^ (average=0.30, range 29-38%). When compared with age-matched normal volunteers, ECV fraction in the HCV-infected individuals was significantly increased (0.30 ±0.03 vs. 0.26±0.03, p=0.0036) (Figure 1). Three of 10 HCV-infected individuals had non-ischemic patterns of gadolinium enhancement on the LGE images (Figure 2). For HCV-infected individuals, markers of inflammation and myocardial damage were within laboratory reference range for all subjects in the CMR cohort, and ECV fraction was elevated regardless of myocardial damage markers or liver fibrosis stage.

**Figure 2.**
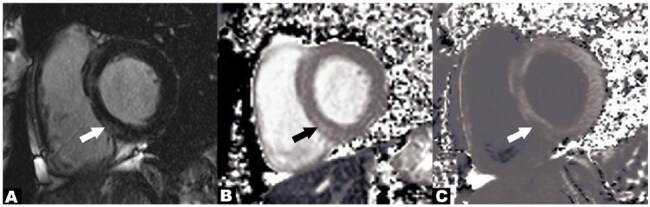
A) Short axis late gadolinium enhancement image shows a midwall pattern of enhancement within the basal septal wall (arrow), compatible with an area of midwall fibrosis. B) Native T1 map and C) post contrast T1 map show a corresponding area of abnormal T1 measurements within the basal septum (arrows).

**Conclusion:**

Our findings suggest that myocardial changes secondary to HCV infection can occur without measurable changes in inflammatory or myocardial biomarkers, and ECV fraction via CMR may be a sensitive screening tool to detect these changes. This has important implications for the necessity of early HCV treatment, since cardiovascular changes can precede the development of advanced liver fibrosis in HCV-infected individuals.

**Disclosures:**

All Authors: No reported disclosures

